# Probing Blood Plasma Protein Glycosylation with Infrared Spectroscopy

**DOI:** 10.1021/acs.analchem.3c03589

**Published:** 2024-02-07

**Authors:** Liudmila Voronina, Frank Fleischmann, Jelena Šimunović, Christina Ludwig, Mislav Novokmet, Mihaela Žigman

**Affiliations:** † Ludwig Maximilian University of Munich, Garching 85748, Germany; ‡ Max Planck Institute of Quantum Optics, Garching 85748, Germany; § Glycoscience Research Laboratory, Genos Ltd., Zagreb 10000, Croatia; ∥ Bavarian Center for Biomolecular Mass Spectrometry (BayBioMS), Technical University of Munich (TUM), Freising 85354, Germany

## Abstract

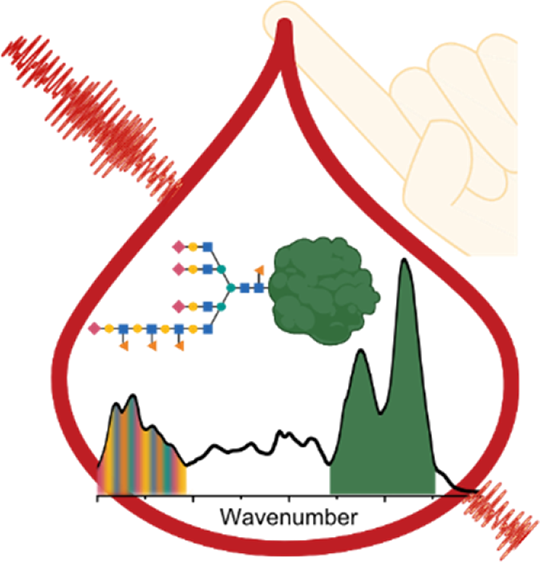

The health state of an individual is closely linked to the glycosylation patterns of his or her blood plasma proteins. However, obtaining this information requires cost- and time-efficient analytical methods. We put forward infrared spectroscopy, which allows label-free analysis of protein glycosylation but so far has only been applied to analysis of individual proteins. Although spectral information does not directly provide the molecular structure of the glycans, it is sensitive to changes therein and covers all types of glycosidic linkages. Combining single-step ion exchange chromatography with infrared spectroscopy, we developed a workflow that enables the separation and analysis of major protein classes in blood plasma. Our results demonstrate that infrared spectroscopy can identify different patterns and global levels of glycosylation of intact plasma proteins. To showcase the strengths and limitations of the proposed approach, we compare the glycoforms of human and bovine alpha-1-acid glycoproteins, which exhibit highly variable global levels of glycosylation. To independently evaluate our conclusions, the glycan moieties of human alpha-1-acid glycoprotein were further analyzed using an established glycomics workflow. Importantly, the chromatographic separation of blood plasma improves the detection of aberrant glycoforms of a given protein as compared to infrared spectroscopy of bulk plasma. The presented approach allows a time-efficient comparison of glycosylation patterns of multiple plasma proteins, opening new avenues for biomedical probing.

## Introduction

Over half of human proteins are decorated by at least one glycan, making glycosylation the most common and complex post-translational modification.^1,2^ The process is non-template-driven; aberrant protein glycosylation accompanies a variety of pathophysiological conditions and may predict several long-term health risks.^3−7^ For instance, even glycoforms of classical, highly abundant plasma proteins show high potential to differentiate cancer from benign diseases of the same organ and to further distinguish between variants and stages of a given tumor.^8−11^ However, the approach is rarely applied in clinical context.^2,12^

This calls for a comprehensive, minimally biased, and robust analytical strategy that would be universally applicable to a wide range of plasma proteins. Here, we put forward vibrational spectroscopy for the analysis of blood glycoproteins. It is inherently suited for the quantitative analysis of glycosylation, since the signals originating from glycans arise at different infrared (IR) spectral ranges than the major signals from the protein backbones.^13^ Therefore, intact proteins can be investigated, eliminating the need for protein digestion or glycan release,^3^ and all types of glycans (N-, O-glycans) can be studied simultaneously. Moreover, IR spectroscopic measurements are experimentally straightforward, with short measurement time, performed at low cost, and highly reproducible.^14−16^ Although the overlap between the absorption bands of different glycan moieties hinders their identification, IR spectra of intact proteins are sensitive to a change in degree of glycosylation and saccharide composition (i.e., micro- and macroheterogeneity).^13,17,18^

Previously, infrared spectroscopy was used to evaluate glycosylation of proteins in pioneering work in 2005–2006,^13,19^ but only recently, the understanding of biological importance of glycosylation prompted further research in this direction.^20−22^ For instance, IR spectroscopy has been proposed as a tool to control glycosylation of therapeutic monoclonal antibodies.^21,22^ Albeit promising, it has not yet, to our knowledge, been applied to proteins derived from human blood plasma.

To detect informative changes in glycosylation patterns of blood proteins, one has to probe their spectroscopic features independently of the contributions of other abundant molecules. Based on existing protocols,^23,24^ we developed a method to separate the abundant plasma glycoproteins into well-defined fractions using ion exchange (IEX) chromatography. We demonstrate the feasibility of the approach by separating bulk plasma into 11 fractions and measuring their IR spectra. Furthermore, we show that our method can be used to resolve various glycoforms of a given protein. As an example, we focus on alpha-1-acid glycoprotein and record IR spectra of different glycoforms for human and bovine alpha-1-acid glycoprotein variants. To independently verify our observations and gain molecular understanding of the differences between the glycoforms of human alpha-1-acid glycoprotein, we involved established glycomics workflow, which includes enzymatic glycan release, glycan labeling, chromatographic separation, and mass spectrometric characterization.

Finally, we model a clinically relevant scenario: as a result of a certain health aberration (disease), the glycosylation pattern of a particular blood plasma protein is altered. In order to detect the disease, one should be able to identify such changes despite the omnipresent biological variability stemming from other molecules. We demonstrate that the IEX separation can reduce the effect of biological variability and thereby advance the limit of detection of aberrant glycoforms close to that in pure water. Provided results thus suggest that the combined IEX+IR workflow can be applied to investigate glycosylation patterns of major plasma proteins in clinical samples.

## Experimental Section

### Chemicals and Reagents

Organic solvents of HPLC grade, sodium chloride, hydrogen chloride, piperazine, bis-tris-propane, and 1,4-dimethylpiperazine at highest available purity were purchased from Sigma-Aldrich GmbH (Taufkirchen, Germany). Ribonucleases from bovine pancreas (CAS-No 9001-99-4)—A with purity above 90% and B with purity above 80%—as well as purified proteins from human plasma (HSA, IgG, alpha-1-antitrypsin, haptoglobin, alpha-1-acid glycoprotein) were also purchased from Sigma-Aldrich GmbH at the highest available purity. Alpha-1-acid glycoprotein from bovine plasma with 99% purity was purchased from the same vendor.

Phosphate-buffered saline (10× PBS), pH 6.6, was prepared in-house. Igepal-CA630, dithiothreitol, 2-methylpiridine borane complex, procainamide hydrochloride (ProA), and dimethyl sulfoxide (DMSO) were purchased from Sigma-Aldrich, St. Louis, Missouri, USA. Glacial acetic acid was produced by Merck, Darmstadt, Germany; acetonitrile (ACN, LC-MS grade) was from Honeywell, USA; sodium dodecyl sulfate (SDS) was from Invitrogen, Carlsbad, California, USA. Peptide-*N*-glycosidase F (PNGase F) was from Promega, Madison, Wisconsin, USA.

### Plasma Sample Collection

The human plasma samples used in the initial experiments were obtained from a monocentric prospective study under research study protocol number 20-199. The included plasma samples of healthy subjects are derived from the Asklepios Biobank for Lung Diseases under project number 333-10 and study protocol number 17-141. Both research protocols were approved by the Ethics Committee of the Ludwig-Maximillian-University (LMU) of Munich and performed in compliance with all relevant ethical regulations, conducted according to Good Clinical Practice (ICH-GCP) and the principles of the Declaration of Helsinki. All participants signed a written informed consent form.

Blood samples were collected, processed, and stored using the same defined standard operating procedures. Venous blood was obtained using Safety-Multifly needles of 21G (Sarstedt) and transferred to 4.9 mL EDTA-plasma tubes Monovettes (Sarstedt). Within 3 h, the samples were centrifuged at 2000 g for 10 min at 20 °C. The samples were manually aliquoted into 500 μL fractions and frozen at −80 °C within 5 h after collection. Directly prior to use, the samples were thawed and centrifuged for 15 min at 15,000*g*.

### SPE and HPLC Workflow

For the solid phase extraction of proteins from crude plasma samples, we used Sep-Pak Accell Plus QMA 3 cc cartridges, with 500 mg of sorbent per cartridge (Waters Corporation). We conditioned the cartridge using buffer A (see below), applied the sample diluted 1:1 with buffer A, washed the cartridge with buffer A, and finally eluted the proteins using buffer B with 500 mM of NaCl. We then exchanged the solvent with buffer A using Amicon Ultra-2 centrifugal filters (10 kDa MWCO, 2 mL sample volume, purchased from Merck, Darmstadt, Germany).

As analytical column, we used YMC BioPro IEX QA with 5 μm particle size, 100 nm pore size, and dynamic binding capacity 110 mg per mL of resin (column material, PEEK; column length, 50 mm; column inner diameter, 4.6 mm). This column has been shown by the manufacturer to efficiently separate human serum proteins, although with salt gradient, as opposed to the pH gradient that we used in this work.

The usable range of pH for this type of chromatographic column spans from 2 to 12 pH units. However, we found that most human plasma proteins elute in the range from 9.0 to 2.8 pH units and, therefore, used this range for the separation. Such broad range of pH values requires a composite buffer with multiple p*K*_a_ points. Based on a previous work,^23^ we produced a buffer that consists of three components: piperazine, bis–tris-propane, and 1,4-dimethylpiperazine, each with two p*K*_a_ points. The concentration of each component was 10 mM. To produce the buffer solutions, all three components were first mixed into water and then the resulting solution was divided into two equal parts. The pH of the first part was adjusted to 9.0 (or 10.5 for the RNase separation) using a 25% HCl solution. Correspondingly, the second buffer was adjusted to pH 2.8 or 3.0 depending on the application. Mixing the two buffers produces a linear pH gradient, as shown in Figure S1.

We used the UltiMate 3000 HPLC system (Thermo Fisher Scientific) equipped with a mobile phase online degasser, a quaternary pump (gradient delay volume 200 μL), an autosampler, a column thermostated compartment, and a diode array detector. The column was kept at 25 °C during protein separation, while the samples were kept at 5 °C. The chromatograms were recorded at 280 nm (at 4 nm width). The gradients employed for each experiment are presented in Figures S2–S5. After the separation, the protein fractions were collected into deep-well plates kept at 5 °C and frozen at −20 °C until further use. After every gradient run, 1 M solution of NaCl was pumped through the column for 5 min.

The protein fractions collected after the IEX separation were thawed at 5 °C. Each fraction contained 400–900 μL of protein dissolved in HPLC buffer. To exchange the buffer to water and concentrate the protein, we employed Amicon Ultra-0.5 Centrifugal Filters (10 kDa MWCO, 0.5 mL sample volume, purchased from Merck, Darmstadt, Germany) according to the manufacturer’s specification.

The protein fractions were characterized by using protein electrophoresis (SDS-PAGE). For that, SERVAGel TG PRIME 4–20% was used with SERVA Triple Color Protein Standard III according to the manufacturer’s specifications.

### LC-MS/MS Proteomics Workflow

The plasma protein fractions collected after IEX, as well as unfractionated plasma proteome samples and plasma samples after SPE extraction, were exchanged with 50 mM NH_4_HCO_3_ buffer and subjected to proteomics analysis. In addition, the eluent between fractions 1 and 2 (labeled A4) and between 10 and 11 (labeled B7) was collected for completeness. For each fraction, 15 μg of total protein amount was used for further processing, as determined by the Bradford assay. All samples were reduced (10 mM DTT, 30 min, 30 °C) and carbamidomethylated (55 mM CAA, 30 min, room temperature). Digestion of proteins was carried out by addition of trypsin (proteomics grade, Roche) at a 1/50 enzyme/protein ratio (w/w) and incubation at 37 °C overnight. Digests were acidified by addition of 0.5% (v/v) formic acid (FA) and desalted using self-packed StageTips (three disks per microcolumn, o̷ 1.5 mm, C18 material, 3M Empore). The peptide eluates were dried to completeness and stored at −80 °C. For LC-MS/MS analysis, all samples were resuspended in 50 μL of 2% acetonitrile and 0.1% FA in HPLC-grade water and 2 μL of sample volume was injected into the mass spectrometer per measurement.

LC-MS/MS measurements were performed on a Dionex UltiMate 3000 RSLCnano system coupled to a Q Exactive HF-X mass spectrometer (Thermo Fisher Scientific). Peptides were loaded onto a trap column (ReproSil-Pur C18-AQ, 5 μm; Dr. Maisch, 20 mm × 75 μm, self-packed) at a flow rate of 5 μL/min in 100% solvent A (0.1% FA in HPLC-grade water). Subsequently, peptides were transferred to an analytical column (ReproSil Gold C18-AQ, 3 μm, Dr. Maisch, 400 mm × 75 μm, self-packed) and separated using a 50 min linear gradient from 4 to 32% of solvent B (0.1% FA in acetonitrile and 5% (v/v) DMSO) in solvent A (0.1% FA in HPLC-grade water and 5% (v/v) DMSO) at a 300 nL/min flow rate. The Q-Exactive HF-X was operated in data-dependent acquisition (DDA) mode, automatically switching between the MS1 and MS2 spectrum acquisition. MS1 spectra were acquired over a mass-to-charge (*m*/*z*) range of 360–1300 *m*/*z* at a resolution of 60,000 using a maximum injection time of 45 ms and an AGC target value of 3e6. Up to 18 peptide precursors were isolated (isolation window 1.3 *m*/*z*), fragmented by high-energy collision-induced dissociation (HCD) using 26% normalized collision energy, and analyzed at a resolution of 15,000, a scan range from 200 to 2000 *m*/*z*, a maximum injection time of 25 ms, and an AGC value of 1e5. Precursor ions that were singly charged, unassigned, or with charge states >6+ were excluded. The dynamic exclusion duration of the fragmented precursor ions was 25 s.

Peptide identification and quantification were performed using MaxQuant (version 1.6.3.4).^37^ MS2 spectra were searched against the human reference protein database from UniProt (UP000005640, download August 2020, 20,353 protein entries), supplemented with common contaminants (built-in option in MaxQuant). Carbamidomethylated cysteine was set as fixed modification, and oxidation of methionine and N-terminal protein acetylation were set as variable modifications. Trypsin/P was specified as the proteolytic enzyme. Precursor tolerance was set to 4.5 ppm, and fragment ion tolerance was set to 20 ppm. Results were adjusted to 1% false discovery rate (FDR) on peptide spectrum match (PSM) and protein level employing a target-decoy approach using reversed protein sequences. The minimal peptide length was defined as seven amino acids, and the “match-between-run” function was disabled.

To quantify the detected proteins per fraction, the “intensity-based absolute quantification” (iBAQ) algorithm was employed.^38^ iBAQ values provide an absolute concentration estimate that can be used to compare the abundances of different proteins present in the same sample. By dividing the iBAQ value of a given protein by the summed iBAQ values of all proteins detected in a given sample, “protein mass fractions” were calculated. The abundances of protein classes IgG, IgA, apolipoproteins, and orosomucoid were calculated as a sum of all identified proteins with the abundance above 0.1% that belong to the corresponding protein class. Only proteins/protein classes that constitute above 5% of the dry protein mass of the fraction were considered separately, while other entries were combined as “Other”.

### UHPLC-MS/MS Glycomics Workflow

The fractions of human alpha-1-acid glycoprotein (ORM) were separated with IEX chromatography (in duplicate) and concentrated to 35–45 μL in 50 mM ammonium bicarbonate, as described above. Each fraction was then subjected to *N*-glycan release, ProA labeling, HILIC-SPE cleanup, and HILIC-UHPLC- ESI-qTOF-MS analysis, processed in triplicates.

First, dried fractions were dissolved in 20 μL of 0.4% SDS (v/v) and 4 μL of 100 mM dithiothreitol and denatured at 95 °C for 5 min. After the mixture was cooled to room temperature, 5 μL of 10× PBS and 20 μL of Igepal-CA630 were added to the samples. *N*-Glycans were released overnight (18 h) at 37 °C with 10 U of PNGase F.

Fluorescent labeling was performed as previously described.^39^ Briefly, *N*-glycans were incubated at 65 °C for an hour with procainamide (43.2 mg/mL) as a fluorescent label and afterward an additional 1.5 h with 2-methylpiridine borane complex (44.8 mg/mL) as a reducing agent. The fluorescent label and reducing agent were prepared in a 25 μL mixture of DMSO and glacial acetic acid (70:30, v/v) separately. HILIC-SPE purification was done prior to HILIC-UHPLC-MS/MS analysis following the protocol published before^40^ using the wwPTFE plate, 0.2 μm, instead of the GHP filter plate, 0.2 μm (Pall Corporation, Ann Arbor, Michigan, USA).

Procainamide-labeled *N*-glycans were separated at a flow rate of 0.4 mL/min with a linear gradient of 65–55% solvent B in 18 min and then isocratic for the next 5 min. The separation was performed on a Waters bridged ethylene hybrid (BEH) glycan chromatography column, 150 × 2.1 mm, 1.7 μm BEH particles, maintained at 50 °C, while samples were loaded on the column under the starting gradient condition of 70% solvent B and maintained at 10 °C before injection. Solvent A was 100 mM ammonium formate, pH 4.4, and solvent B was ACN.

Fluorescence detection signals and MS/MS spectra were recorded for each sample. The wavelengths for excitation and emission were 310 and 370 nm, respectively. MS parameters were set as described previously^39^ with the difference in mass range from 100 to 4000 *m*/*z* and a frequency of 0.5 Hz. *N*-Glycan structures in peaks were annotated in Bruker DataAnalysis 4.1. and determined from sum spectra created for 35 chromatographic peaks based on their retention time. The *N*-glycan composition for each chromatographic peak was proposed based on features detected in sum spectra.

### FTIR Measurements and Processing of the Spectra

The collected and processed protein fractions were measured in the liquid phase with an automated FTIR device (MIRA Analyzer, Clade GmbH, Esslingen am Neckar, Germany) with a flow-through transmission cuvette (CaF_2_ with 8 μm path length). The spectra were acquired with a resolution of 4 cm^–1^ in a spectral range between 950 and 3050 cm^–1^ but truncated to 950–3000 cm^–1^. “Negative” absorption occurs in the wavenumber region 1850–2150 cm^–1^ because the hydrated sample contains less water than the reference (pure water) and was corrected for, as described previously.^41^ The same wavenumber region was subsequently utilized to compensate for baseline drifts. The spectra were further vector-normalized to emphasize the differences in their shapes as opposed to their intensity.

### Calculation of the Spectroscopic Global Glycosylation Index

Following the algorithm proposed by Derenne et al.,^18^ we calculated the global glycosylation level based on the spectroscopic data. First, we recorded and processed the FTIR absorption spectra of the protein or the glycoform as described above. Second, we normalized the spectra to the absorption of the protein backbone—to the area under the absorption spectrum between 1476 and 1718 cm^–1^. Next, we integrated the spectral range between 1000 and 1179 cm^–1^ with the straight line connecting the two end points as baseline; this is the spectroscopic global glycosylation index, as it reflects the ratio between the absorption of the glycan chains and the protein backbone or, in other words, the global level of glycosylation. Note that the protein backbone exhibits certain, although weak, absorption in the region 1000–1200 cm^–1^ and therefore the global glycosylation index is positive even in the absence of glycosylation. For example, the spectroscopic global glycosylation index of HSA purified from human serum is 0.09, although the proportion of glycated albumin in healthy persons is between 1 and 10%.^29^

### Distinguishing RNase A from RNase B: Experimental Design

For every protein concentration, we measured the 40 replicas of RNase A and B in pure water, alternating the type of RNase (to avoid bias related to the instrument drift). After spectrum processing, we calculated the global glycosylation indexes from each spectrum, the average difference in the global glycosylation index between RNase A and B, and the standard deviation for this difference. The results are presented in Figure S11 (black dots). Note that the measured difference in the spectroscopic global glycosylation index—0.1 or 10%—is close to the difference in global glycosylation of the two proteins reported in the literature in terms of mass (9%),^33^ despite potentially different infrared absorption cross sections of the glycans and the protein backbone.

In the next step, we randomly chose 10 blood plasma samples of reportedly healthy individuals and prepared 80 μL aliquots of those. In five randomly selected samples, we spiked 20 μL of RNase A in a given concentration and into another five -- of RNase B, and measured the FTIR absorption spectra. Here, we also observe a certain difference in the global glycosylation indexes but much smaller than in pure protein solution because the absorption signal is dominated by other proteins in blood plasma.

Finally, we prepared the samples in the same way as described above, but prior to the FTIR measurement, we separated the RNase-containing fraction using IEX chromatography and processed it as described above for plasma proteins. The example chromatograms of RNase A and B spiked into human plasma are shown in Figure S12 and the difference in global glycosylation indices between RNase A and B in Figure S11, red dots.

### The Effect Size Estimation

We calculated the effect size (the “measure of discrimination” in the original publication^34^) as a measure of how significant the difference between spectroscopic global glycosylation indices for RNase A and B at various concentrations is (Figure 4b):
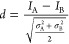
where *d* is the effect size for a given protein concentration and experimental conditions, *I*_A_ is the average spectroscopic global glycosylation index for RNase A for this concentration at these experimental conditions, *I*_B_ is the same for RNase B, and σ_A_ and σ_B_ are their standard deviations.

## Results and Discussion

### Infrared Molecular Fingerprinting of Blood Plasma Glycoprotein Fractions

Ion exchange and, in particular, strong anion exchange chromatography are frequently used for protein separation and purification due to the high binding capacity and recovery of biomolecules.^25^ For IR spectroscopic measurements, it is important to minimize the concentration of substances that would interfere with downstream application. In particular, we chose to use a pH gradient,^23,26^ keeping the total concentration of salts in the buffer at 30 mM, while in commonly used salt gradient, their concentration rises to at least 500 mM.^24,27^ In order to cover the broad variety of pI values typical for the proteins in a crude blood plasma sample, a previously described custom buffer solution was used.^23^ We further minimized the concentration of salts by choosing only three buffer components with two p*K*_a_ points each. The resulting buffers A and B are adjusted to pH values 9.0 and 3.0, respectively, and the pH value changes linearly with the mixing ratio (Figure S1), a prerequisite of a robust IEX method.^23^

In order to prolong the lifetime of the ion exchange chromatographic column and improve the reproducibility, we introduced solid phase extraction (SPE) prior to chromatographic separation. We chose strong anion exchange (SAX)-based SPE, which is the same type of interaction as that employed in the chromatographic step. Such an approach ensures that the proteins that tend to precipitate as the pH changes from acidic to basic would do so already at the SPE step, preventing the clogging of the analytical column.

**Figure 1:**
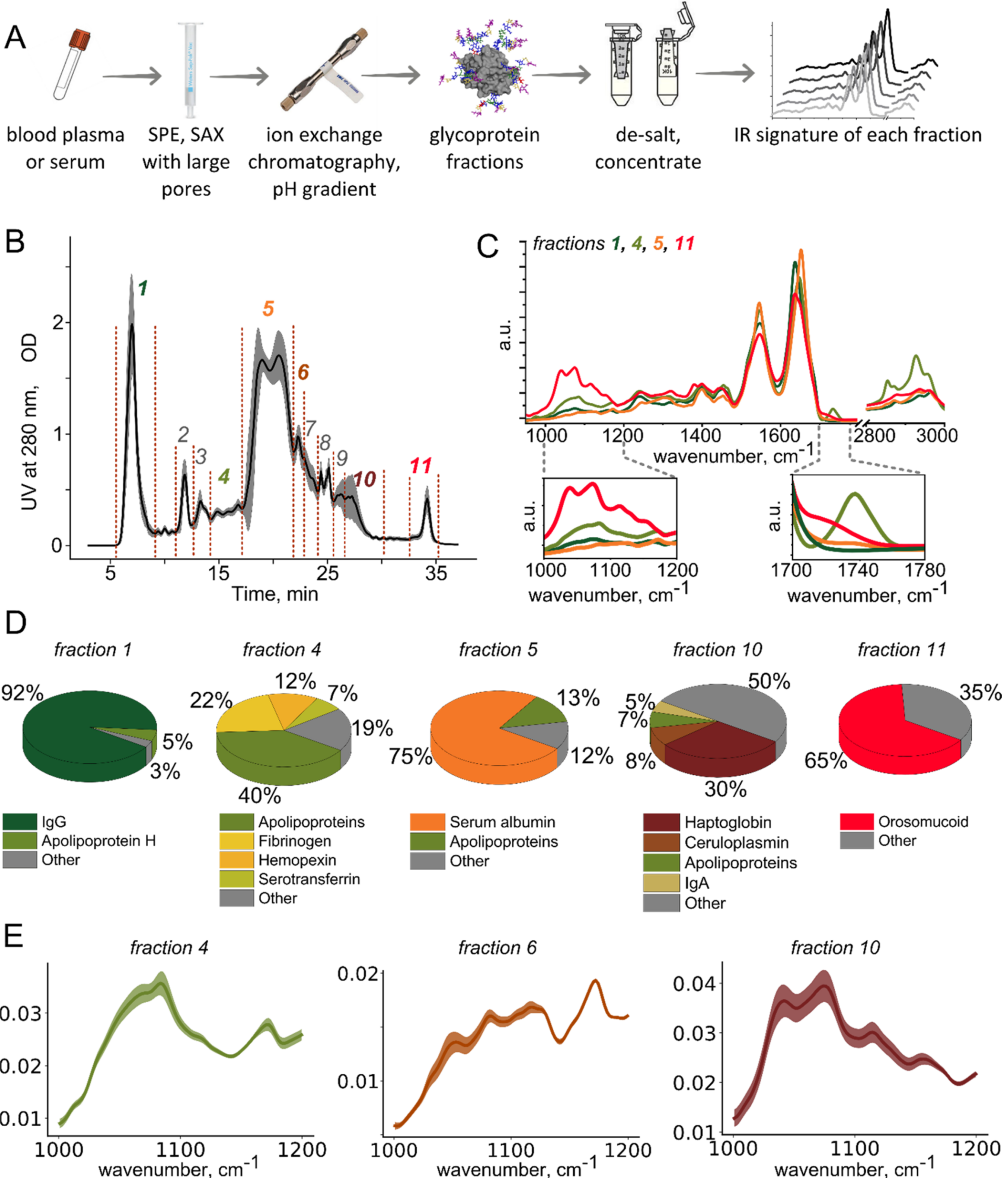
(A) Schematic of the developed and applied analytical workflow: infrared molecular fingerprinting of glycoprotein fractions obtained from crude blood plasma via SPE extraction, ion exchange (IEX) chromatographic separation, desalting, and concentration. (B) Average chromatogram of crude plasma samples from healthy individuals obtained using UV detection at 280 nm. Eleven typically collected fractions are labeled with numbers. (C) Examples of the infrared spectra of the protein fractions 1, 4, 5, and 11. (D) The composition of selected fractions measured via MS-based proteomics. (E) Between-person variability of the infrared spectra in the carbohydrate region, measured in a group of 25 healthy individuals.

We used a set of 25 samples from asymptomatic subjects to demonstrate the method’s performance and to roughly assess the between-person variability (see Table S1 for the cohort description). First, typical chromatograms of blood plasma were collected with the peaks corresponding to human serum albumin and the most abundant plasma glycoproteins (Figure 1b). While the number of fractions collected after IEX can be adjusted, we established collection of 11 fractions—providing a compromise between separating all prominent peaks in the chromatogram and obtaining sufficient amount of material to perform spectroscopic measurements of every fraction. The UV-based chromatogram provides information on the abundances of major proteins and the ratios of the proteoforms with different acidity. We observe the highest relative between-person variability in fractions 9 and 10 (Figure 1b, shaded area). Notably, not only do the relative abundances of the peaks differ here but the shape of the chromatogram as well. At the same time, the position of other major peaks remains constant in the sample set that we investigated.

In order to characterize the degree of chromatographic separation, we first performed SDS-PAGE analysis of the fractions (Figure S6). Some fractions, such as fractions numbers 1, 2, 5, and 11, are dominated by a single protein, while other fractions exhibit—as expected—more different proteins as evident in a number of lanes with similar intensities.

To further quantify the composition of the fractions, we profiled them with mass spectrometry-based proteomics. In brief, the proteins were denatured and digested with trypsin and the resulting peptides were analyzed with LC-MS/MS. Figure 1d demonstrates the revealed protein compositions for specific fractions (see Table S2 for all fractions). For instance, more than 60% of the protein dry mass in fractions 1, 2, 5, and 11 can be attributed to a single protein class, while fractions 8 and 9 contain seven to eight components with abundances over 5%. The category “other” encompasses all proteins with abundances below 5%, of which fraction 10 contains the highest percentage—almost half. This may be the reason for the high between-person variability observed in fraction number 10 (see Figure 1b). In general, our method combines the benefits of targeted analysis of certain proteins (e.g., immunoglobulin G, serotransferrin, orosomucoid, and apolipoproteins) with the advantages of molecular fingerprinting that aims to cover a broader range of molecular types.

Chromatographic separation unavoidably dilutes the samples, deteriorating the signal-to-noise ratio of any IR protein signatures. To combat that, we use ultrafiltration with a 10K molecular weight limit to desalt and concentrate the samples (Figure 1a, see also Experimental Section). Conveniently, the volume of the protein sample after the reverse spin of a filter with a vertical membrane corresponds to the minimal volume required for our spectroscopic measurements (30–50 μL). The final concentration of a glycoprotein during IR measurement is thus comparable to its concentration in the initial plasma sample, ensuring a sufficient signal-to-noise ratio. Importantly, ultrafiltration preserves the native-like state of the proteins.^28^

In the next step, infrared absorption spectra of collected fractions were measured (Figure 1c, see Figure S7 for the spectra of all fractions). The spectral regions between 1000 and 1180 cm^–1^ (so-called “carbohydrate region”) and 1700–1780 cm^–1^ reflect the details of the post-translational modification of the proteins.^13^ Serum albumin, which dominates fraction 5, is not glycosylated and it is nonenzymatically glycated only to a low degree.^29^ This protein thus exhibits little absorption in the carbohydrate region, while the high global glycosylation level of orosomucoid 1 (41% of protein weight^9^) leads to the strong absorption bands at 1040, 1075, and 1116 cm^–1^ in fraction 11. The overall level of glycosylation of immunoglobulin G (fraction 1) is only 2–3%,^30^ which is reflected in the corresponding IR spectrum. The degree of sialylation is low for IgG; therefore, virtually no absorption is observed between 1700 and 1780 cm^–1^. While the absorption spectra of these fractions overlap well with the spectra of corresponding purified standards (Figure S8), such comparison is not straightforward for fraction 4, where multiple proteins coelute (Figure 1d). Notably, this fraction exhibits strong absorption peaks at 1740, 2854, and 2927 cm^–1^, due to the presence of lipoproteins.

We further addressed the variability in the IR spectra of the collected fractions. Specifically, the high variability in the carbohydrate region of the spectrum is informative for assessing variations in the glycosylation patterns and the degree of glycosylation. The most variable IR spectra with their standard deviation per wavenumber are presented in Figure 1e. Fraction number 10 demonstrates high variability in terms of protein composition (Figure 1b), and therefore, it is expected to be highly variable in terms of IR spectra as well. However, fractions 6 and 9 are especially variable qualitatively, in terms of the shape of their infrared spectra, making them promising candidates for further investigations in clinical context. The smallest relative between-person variability is observed for fraction 5 (Figure S9), most likely because this fraction is dominated by nonglycosylated serum albumin.

### Glycosylation Patterns of Human and Bovine Alpha-1-Acid Glycoproteins

**Figure 2:**
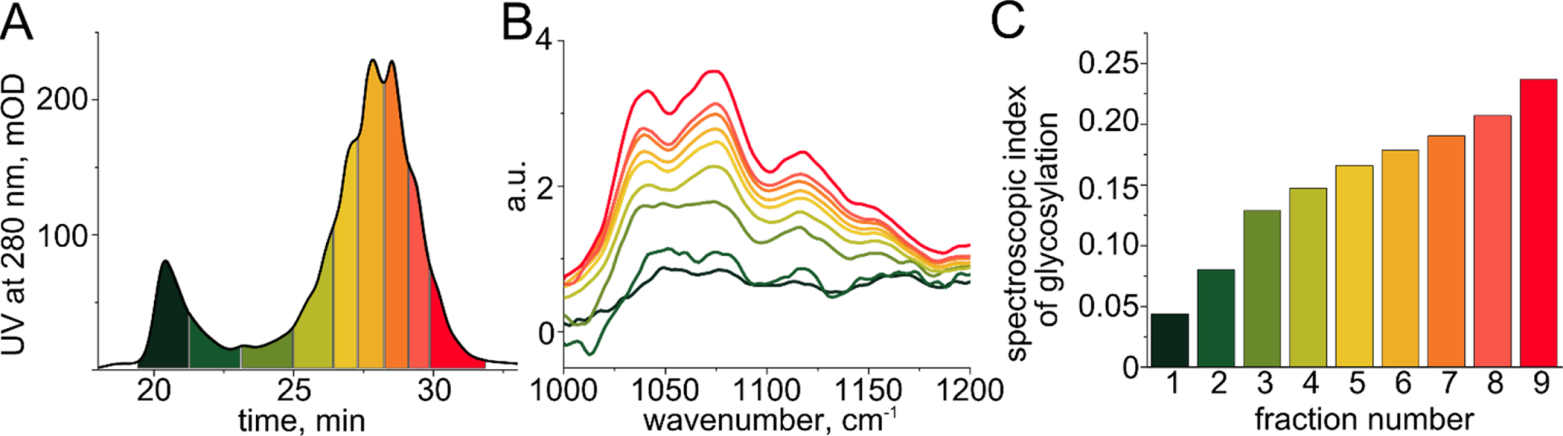
(A) Chromatographic separation of bovine alpha-1-acid glycoprotein with color-coded collected fractions. (B) IR absorption spectra of each protein fraction in the carbohydrate spectral region. (C) Spectroscopic global indices of glycosylation19 (the ratio between glycan and protein backbone absorption) over different fractions with increasing elution times.

**Figure 3:**
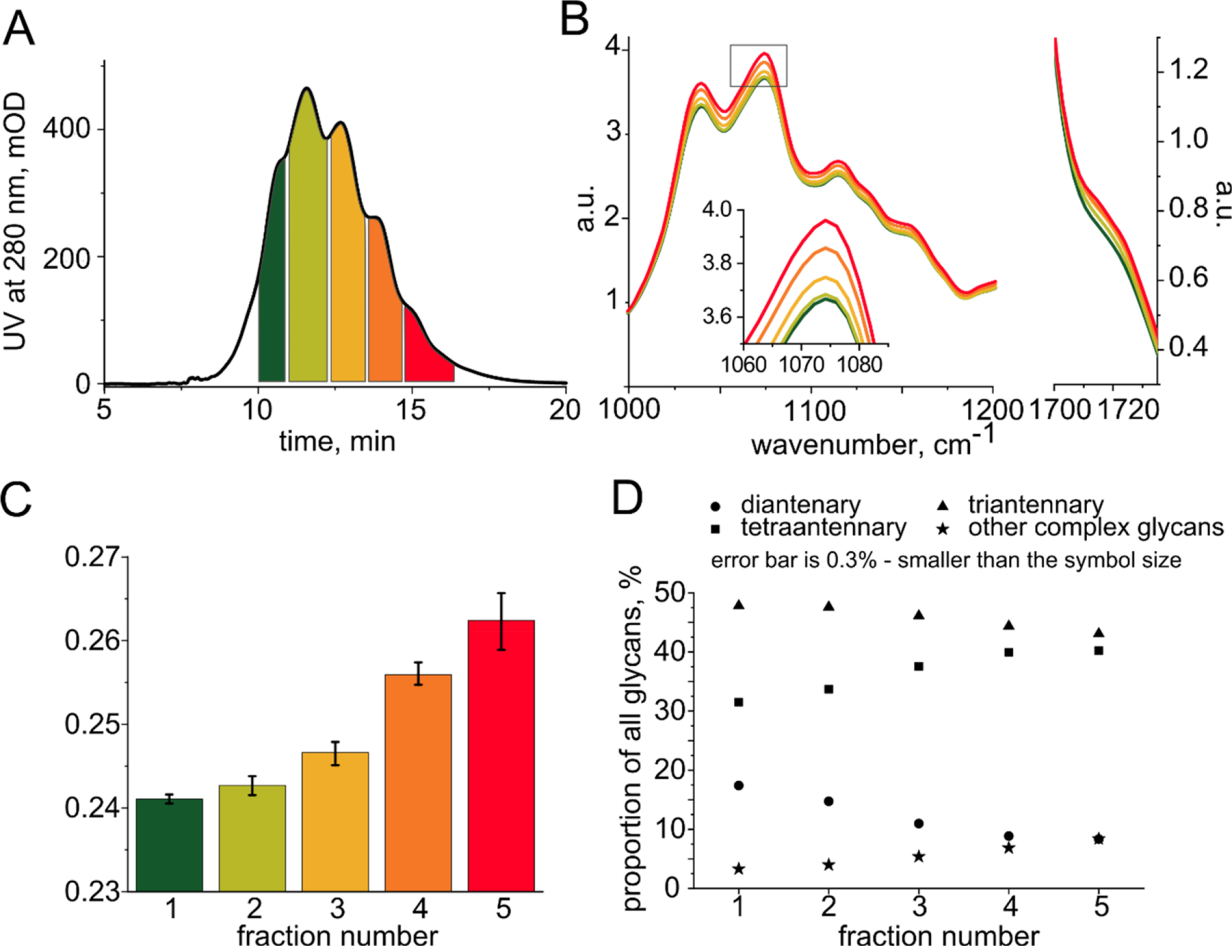
(A) Chromatographic separation of human alpha-1-acid glycoprotein with color-coded collected fractions. (B) IR absorption spectra of each protein fraction. (C) Spectroscopic global indices of glycosylation (the ratio between glycan and protein backbone absorption) in different fractions with increasing elution times. (D) The proportion of di- and tri-antennary glycans decreases with elution time, while the proportion of complex glycans increases, contributing to the growing total mass of the glycans.

Having established that various blood plasma proteins exhibit characteristic IR signatures that reflect their post-translational modifications, we next assessed whether the newly combined IEX+IR analytical workflow has the capacity to disentangle finer details of the glycosylation patterns, for instance, unravel IR signatures that would be specific to different glycoforms of the same protein. We used purified alpha-1-acid glycoprotein (orosomucoid, ORM) from bovine and human plasma as two examples of highly glycosylated and heterogeneous biomolecules.^31^ For the optimal IEX separation of each protein, the pH gradients were adjusted according to their pI values (Figures S3 and S4). After IEX separation (Figures 2a and 3a), we collected each chromatographic peak into a separate fraction and recorded their IR spectra (Figures 2b and 3b). The spectra were normalized to the absorption of the protein backbone (1476–1718 cm^–1^),^18^ and spectroscopic global indices of glycosylation were computed as the integral absorption in the carbohydrate region (1000–1180 cm^–1^), following the definition proposed by Derenne et al.^18^ (see Experimental Section). We find that the spectroscopic global indices of glycosylation consistently grow with elution time (Figure 2c and 3c), suggesting that heavily glycosylated proteoforms are more acidic than those with a lower global degree of glycosylation and therefore elute later in time.

Given that the observed variations in human alpha-1-acid glycoprotein IR spectra are relatively small, we sought to confirm whether they arise from the consistent changes in the glycosylation pattern and to explain these differences at the molecular level. To that end, we employed an independent UHPLC-HILIC/MS-MS-based glycomic workflow (see Experimental Section). The spectroscopic global index of glycosylation refers to the infrared absorption of glycans relative to that of proteins, which, in turn, is proportional to their concentrations. Each of the fractions was thus profiled for the total alpha-1-acid glycoprotein glycome.

The glycans from a predefined amount of protein were released from the protein fractions, labeled, purified, and analyzed using HILIC-UHPLC. The resulting chromatogram consists of 35 peaks (Table S3), corresponding to different *N*-glycans.

Detailed analysis reveals that the difference in the glycosylation patterns of the protein fractions lies in the changing proportions between the glycan structures, which glycomic workflow quantifies with high precision (Figure 3d, see also Figure S10). In particular, the proportion of di- and tri-antennary glycans significantly decreases with elution time, while the proportion of tetra-antennary and other complex glycans exhibits the opposite trend. Therefore, in this case, the infrared absorption spectra reflect the change in the complexity of the glycan structures (microheterogeneity).

In general, using the example of bovine and human alpha-1-acid glycoproteins, we demonstrate that IR spectroscopy allows for an overview of the glycoforms of a given protein and for a direct comparison of the glycosylation patterns. Although the amino acid sequences of bovine and human proteins are not identical, the protein backbone absorption in the glycan region of the infrared spectrum is low, allowing for semiquantitative comparison between two species. First, the human alpha-1-acid glycoprotein has a higher global degree of glycosylation than the bovine one. Second, the glycoforms of bovine glycoprotein are in general more heterogeneous than that of the human protein, although the total number of distinct glycoforms in human alpha-1-acid glycoprotein is estimated to reach 100 in native-MS experiments.^31^ In addition, the bovine glycoprotein has a distinct glycoform that exhibits a particularly low degree of glycosylation (peak 1 in Figure 2a). A question arises: what is the biological role of this low-glycosylated glycoform, and why is it present in the bovine blood plasma but not in human? Generally, such qualitative differences could also be identified within the human population and lead to important breakthroughs in understanding of protein glycosylation functions and regulation. Although the carbohydrate chains derived from bovine and human alpha-1-acid glycoprotein proteins have been analyzed previously,^35^ the conclusions listed above could only be made on the intact protein level.^27^ At the same time, comparison with the UHPLC-HILIC-MS/MS glycomic workflow reveals a limitation of IR spectroscopy as a detection method: although the proportion of complex glycans changes significantly with elution time (Figure 3d), these changes in the glycan composition remain too subtle to significantly alter the shape of the IR spectrum, affecting mostly its intensity (Figure 3b). The more substantial differences in the glycosylation patterns of blood plasma glycoproteins are nonetheless observable.

### Circumventing the Unrelated Biological Variability to Observe the Glycosylation of the Protein of Interest

**Figure 4:**
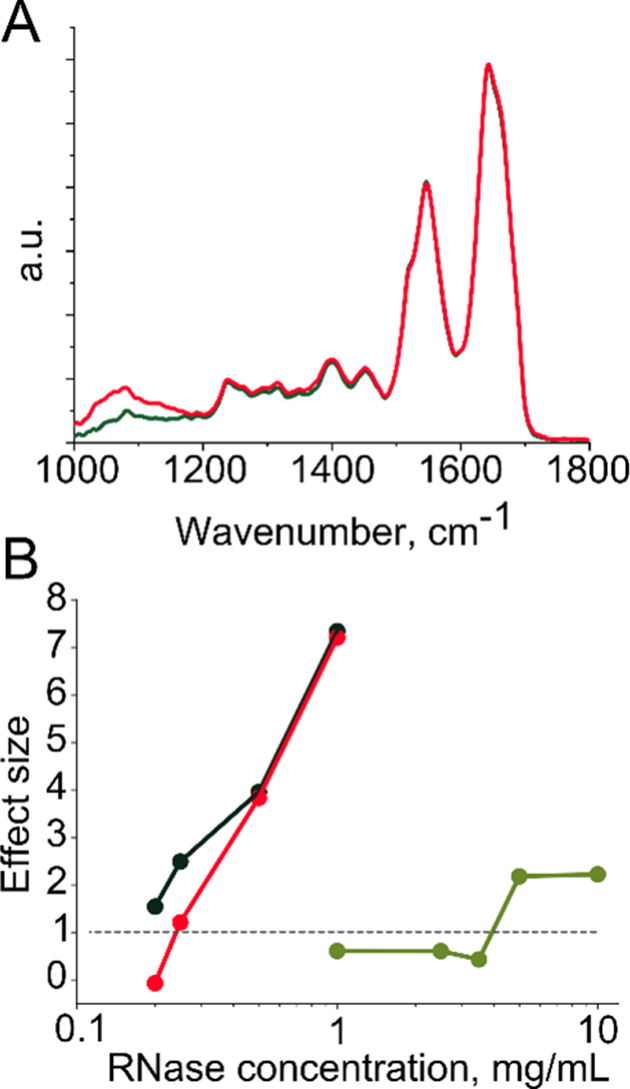
(A) Comparison between the infrared absorption spectra of RNase A (green) and RNase B (red) normalized to the absorption of the protein backbone. The difference in the global degree of glycosylation is visible in the 1000–1200 cm^–1^ region of the spectrum. (B) The ability to distinguish RNase A from RNase B, defined as the standardized effect size as a function of RNase concentration: black, RNase A and RNase B diluted in pure water; green, RNase A and RNase B spiked into blood plasma and measured directly, in bulk; red, RNase A and RNase B spiked into blood plasma and separated by IEX prior to IR measurement.

Changes in the protein glycosylation patterns reflect the health state of an individual^3−7^ and may thus be informative to detect disease. At the same time, the composition of blood plasma of individuals and therefore the infrared spectra of its fractions are intrinsically variable.^32^ Most of this variation stems from the between-person differences that do not correlate with a disease but simply reflect the individual phenotypes. Based on the previous research, the information about certain pathophysiological states is encoded in the glycosylation patterns of a limited number of proteins. To model such situation, we used two proteins, ribonucleases (RNase) A and B, that differ only in their glycosylation level: type A is not glycosylated, while the degree of glycosylation for type B is 9%,^33^ as illustrated by Figure 4a. First, we measured at which concentration the two types of RNase can be distinguished in water: this provides an estimate for the sensitivity of the IR absorption measurement itself (Figure 4b, black points). From each measured IR spectrum, we computed the spectroscopic global glycosylation index and used the standardized effect size as a measure of observed difference between the two RNase glycoforms (see Experimental Section).^34^ When the effect size equals 1, the measurement variability is of the same order of magnitude as the difference in the glycosylation levels; the higher the effect size, the easier it is to distinguish between the two glycoforms. As expected, the effect size grows monotonously with the RNase concentration in water (Figure 4b, black points), since the experimental variability comes from the IR absorption measurement here and only weakly depends on the RNase concentration.

Next, we spiked the blood plasma of healthy individuals with equal amounts of either RNase A or B and measured the resulting IR absorption spectra. Since the variety of molecules contained in blood plasma produce a strong and highly variable spectral background, the concentration at which RNase A can be distinguished from B is significantly higher than in pure water (Figure 4b, green points). The effect size remains low, even when the concentration of RNase in the sample is above 10% of the total protein concentration.

Finally, we aimed to reduce the effect of biological variability by separating the RNase from the rest of the proteins in the plasma samples using IEX. Given the relatively high pI of RNase A and B, we slightly modified the typical plasma separation protocol by increasing the pH of buffer A from 9.0 to 10.5 and shortening the gradient (Figures S5 and S12). The two RNase types coelute, and their separation is not readily possible using IEX alone. After the IEX separation, we collected the fraction containing RNase, desalted it, and measured its IR absorption spectrum. We observe that the minimum RNase concentration at which its glycoform can be determined (Figure 4b, red points) is similar to the detectable concentration when dissolved in pure water (Figure 4b, black points), indicating almost complete elimination of biological variability. Correspondingly, the effect size increases compared with the measurement of RNase A and B when spiked into plasma. We conclude that despite the analytical variability that the sample processing might introduce, IEX separation combined with ultrafiltration helps to reveal the details of the glycosylation pattern of a specific protein within molecularly complex blood plasma.

## Conclusions

To summarize, we lay out a simple, label-free, and universal workflow to study glycosylation of blood plasma proteins that could be extended to medical scenarios. Importantly, the IEX separation with the pH gradient is flexible and facilitates two possibilities: either isolation of a protein of interest or screening across protein classes, depending on the research question. Moreover, a variety of glycoforms can be studied and compared, screening for phenotype aberrations. Due to the low cost and time efficiency of IR spectroscopy, the IEX+IR approach has the potential to leverage glycoform analysis to be more accessible and possibly contribute to clinical applications (e.g., in vitro plasma diagnostics).

Fourier transform infrared spectroscopy in its conventional, broad implementation is reproducible and brings along the ease of experimentation. At the same time, its sensitivity is lower than recently developed spectroscopic configurations such as laser-based field-resolved spectroscopy (FRS), where the dynamic range is increased by at least an order of magnitude compared to Fourier transform spectrometers.^36^ In principle, the combination of IEX with FRS could provide access to less abundant yet informative glycosylated proteins in complex organic matrices.

Most importantly, the presented workflow allows for an unbiased characterization of glycosylation patterns in human plasma samples. If verified to be condition-specific, it could be suited for the detection of aberrant glycoforms in blood-based human samples, which could in turn lead to new disease detection approaches.

## Data Availability

The mass spectrometric raw files as well as the MaxQuant output file have been deposited to the ProteomeXchange Consortium via the PRIDE partner repository and can be accessed using the identifier PXD046796.

## Supporting Information Available

pH of the mixtures of buffers A and B; the HPLC gradient used for plasma separation; the HPLC gradient used for bovine ORM separation; the HPLC gradient used for human ORM separation; the HPLC gradient used for RNase separation; SDS-PAGE characterization; infrared absorption spectra of all fractions; comparison with the purified proteins; between-person variability in all fractions; the change in the level of glycosylation of alpha-1-acid glycoprotein; difference between glycosylation indexes of RNase B and RNase A; chromatographic separation of blood plasma samples with spiked RNase; the cohort for the between-person variability estimation; the composition of protein fractions; and glycan compositions observed for alpha-1-acid glycoprotein (PDF)
