# Supplementary material for: Probing Blood Plasma Protein Glycosylation with Infrared Spectroscopy
Source: Anal Chem. 2024 Feb 7;96(7):2830–9. doi: 10.1021/acs.analchem.3c03589 (PMC10882574; doi:10.1021/acs.analchem.3c03589)
Supplement: Supplementary file 1 — ac3c03589_si_001.pdf [file ac3c03589_si_001.pdf]

Supporting Information: Probing Blood Plasma Protein Glycosylation With Infrared Spectroscopy.

Liudmila Voronina,[*,†,‡] Frank Fleischmann,[†,‡] Jelena Šimunović,[¶] Christina Ludwig,[§] Mislav Novokmet,[¶] and Mihaela Žigman[*,†,‡]

†*Ludwig Maximilian University of Munich, Garching, 85748 Germany*

‡*Max Planck Institute of Quantum Optics, Garching, 85748 Germany*

¶*Glycoscience Research Laboratory, Genos Ltd., Zagreb, 10000 Croatia*

§*Bavarian Center for Biomolecular Mass Spectrometry (BayBioMS), Technical University of Munich (TUM), Freising, 85354 Germany*

E-mail: liudmila.voronina@mpq.mpg.de; mihaela.zigman@mpq.mpg.de

Figures

S1 pH of the mixtures of buffers A and B S-3
S2 The HPLC gradient used for plasma separation S-3
S3 The HPLC gradient used for bovine ORM separation S-4
S4 The HPLC gradient used for human ORM separation S-4
S5 The HPLC gradient used for RNase separation S-4
S6 SDS-PAGE characterization S-5
S7 Infrared absorption spectra of all fractions S-6
S8 Comparison with the purified proteins S-7
S9 Between-person variability in all fractions S-8
S10 The change in the level of glycosylation of alpha-1-acid glycoprotein S-9
S11 Difference between glycosylation indexes of RNase B and RNase A S-9
S12 Chromatographic separation of blood plasma samples with spiked RNase . . S-10

Tables

S1 The cohort for the between-person variability estimation S-3
S2 The composition of protein fractions S-11
S3 Glycan compositions observed for alpha-1-acid glycoprotein S-12

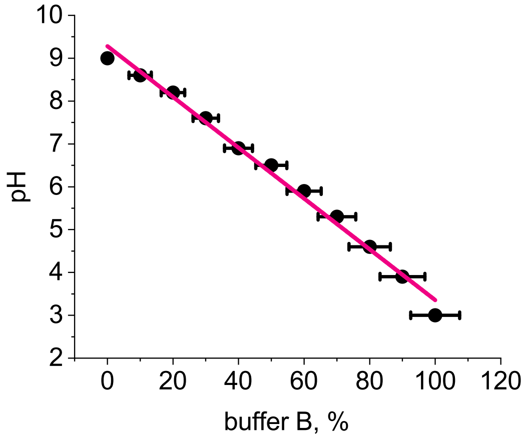

Figure S1: pH of the mixture of buffers A (pH=9.0) and B (pH=3.0) as a function of relative concentration of buffer B. The line shows the linear fit of the data.

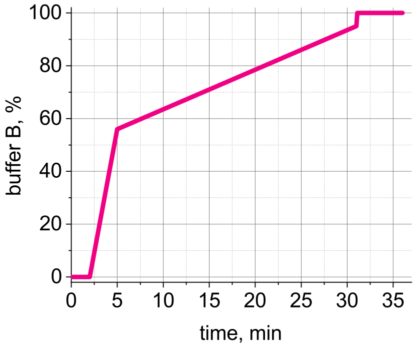

Figure S2: The HPLC gradient used for the separation of blood plasma proteins. Buffer A - pH=9.0, buffer B - pH=3.0.

Table S1: Characteristics of the cohort used for the between-person variability estimation.

Number of samples	25
age	66±11
female	36%
BMI	26±5
diabetes	no
high blood pressure	52%
cancer	no
ethnicity	white
active smokers	24%

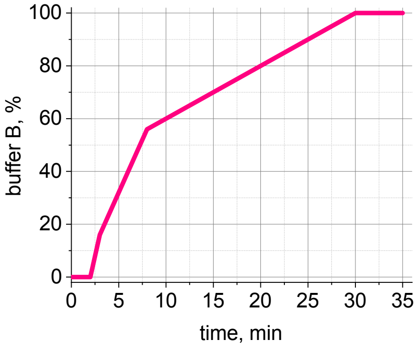

Figure S3: The HPLC gradient used for the separation of glycoforms of bovine alpha-1-acid glycoprotein. Buffer A - pH=9.0, buffer B - pH=2.8.

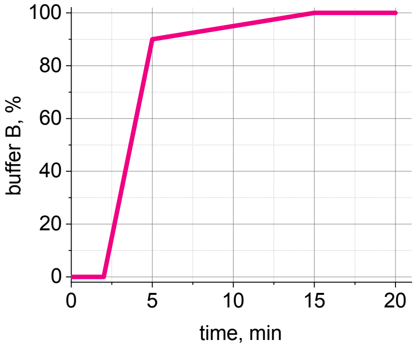

Figure S4: The HPLC gradient used for the separation of glycoforms of human alpha-1-acid glycoprotein. Buffer A - pH=9.0, buffer B - pH=2.8.

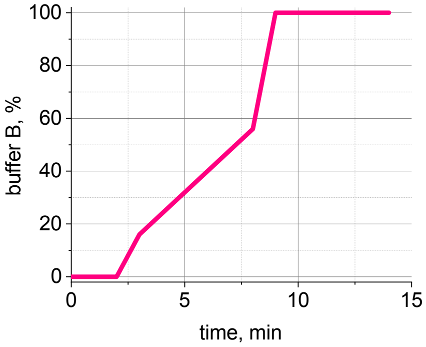

Figure S5: The HPLC gradient used for the separation of RNase spiked into human plasma. Buffer A - pH=10.5, buffer B - pH=2.8.

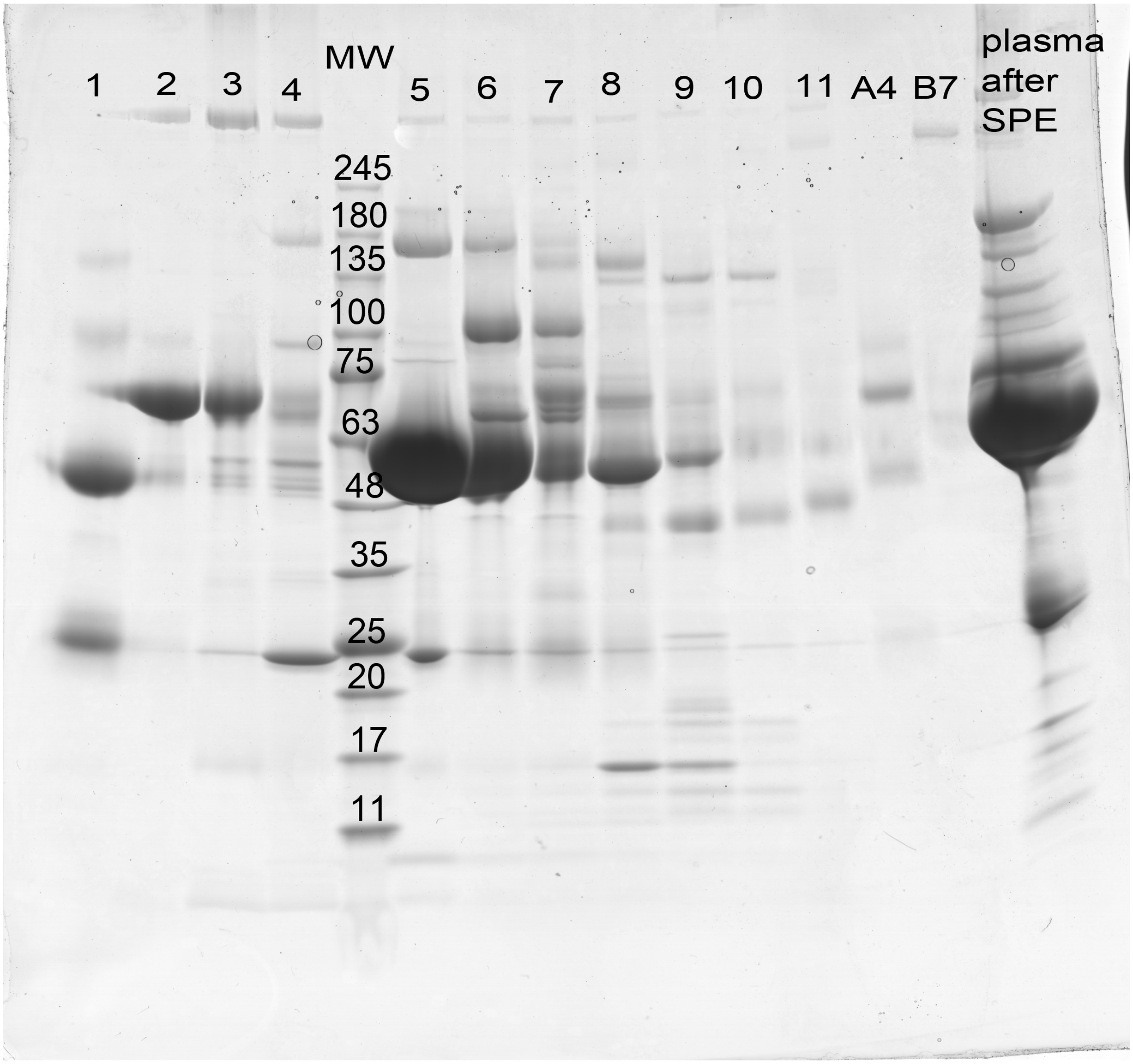

Figure S6: SDS-PAGE characterization of the fractions separated with IEX. The numbers labeling the fractions correspond to Figure 1b. Lane 5 - standard protein mixture with the corresponding molecular weights; lane 15 - the blood plasma after SPE extraction. Fraction A4 is collected between fractions 1 and 2, fraction B7 - between 10 and 11.

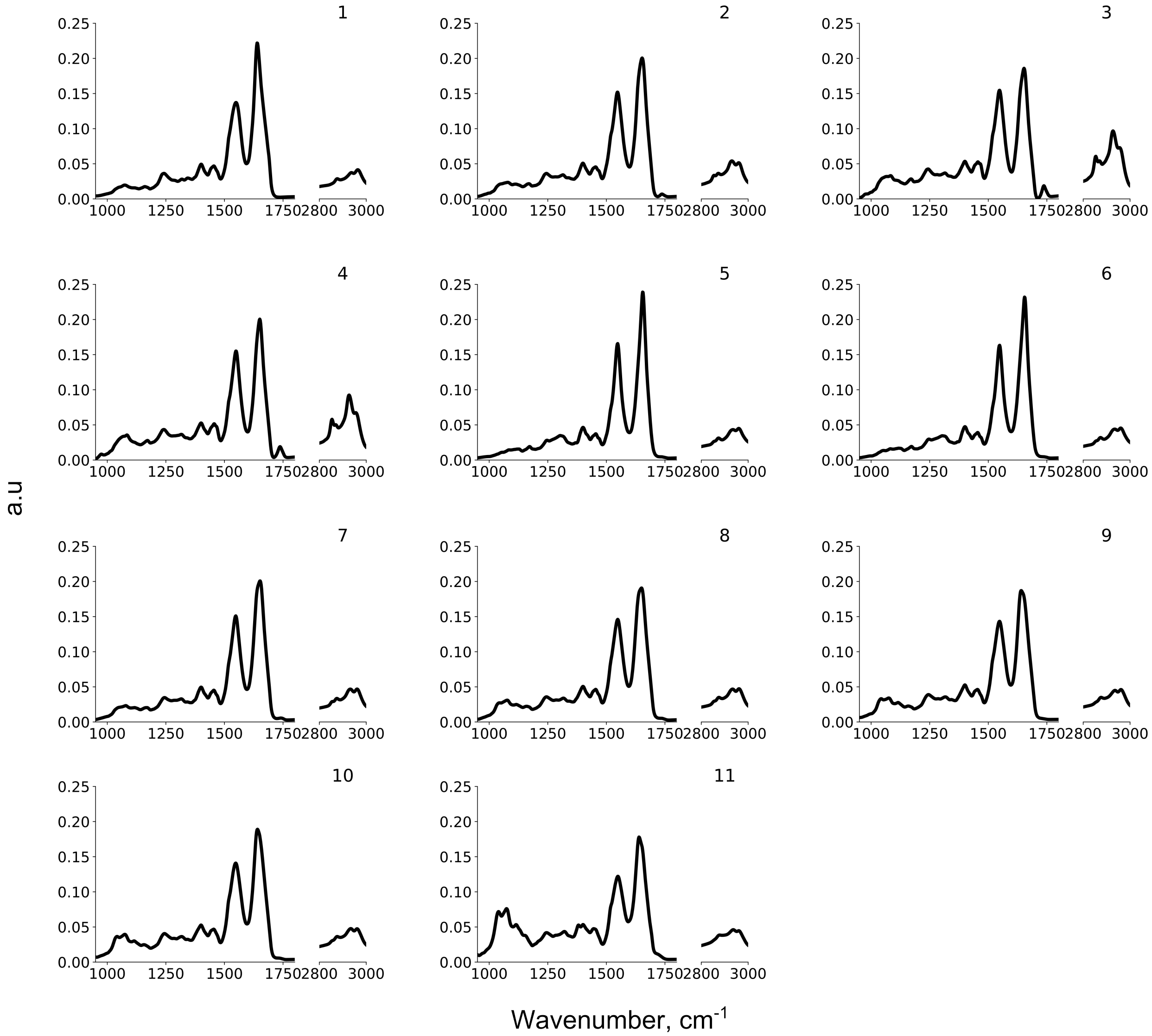

Figure S7: Infrared absorption spectra of all fractions collected from crude plasma samples. The numbers labeling the fractions correspond to Figure 1b.

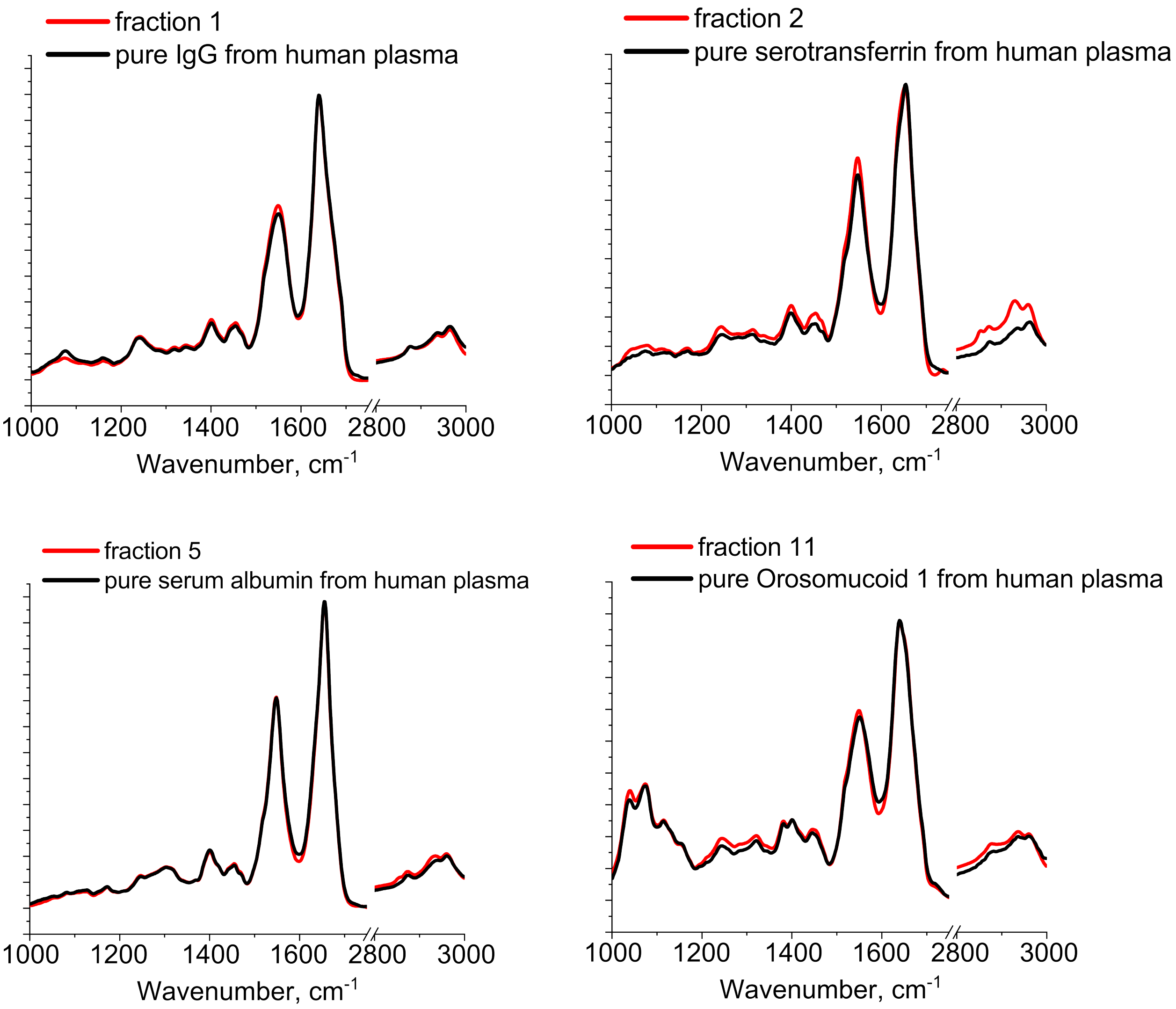

Figure S8: Infrared absorption spectra of the fractions collected from crude plasma samples compared to the infrared spectra of the corresponding purified proteins, purchased from a commercial provider. Only fractions with over 60% of a certain protein are shown (see Table S2).

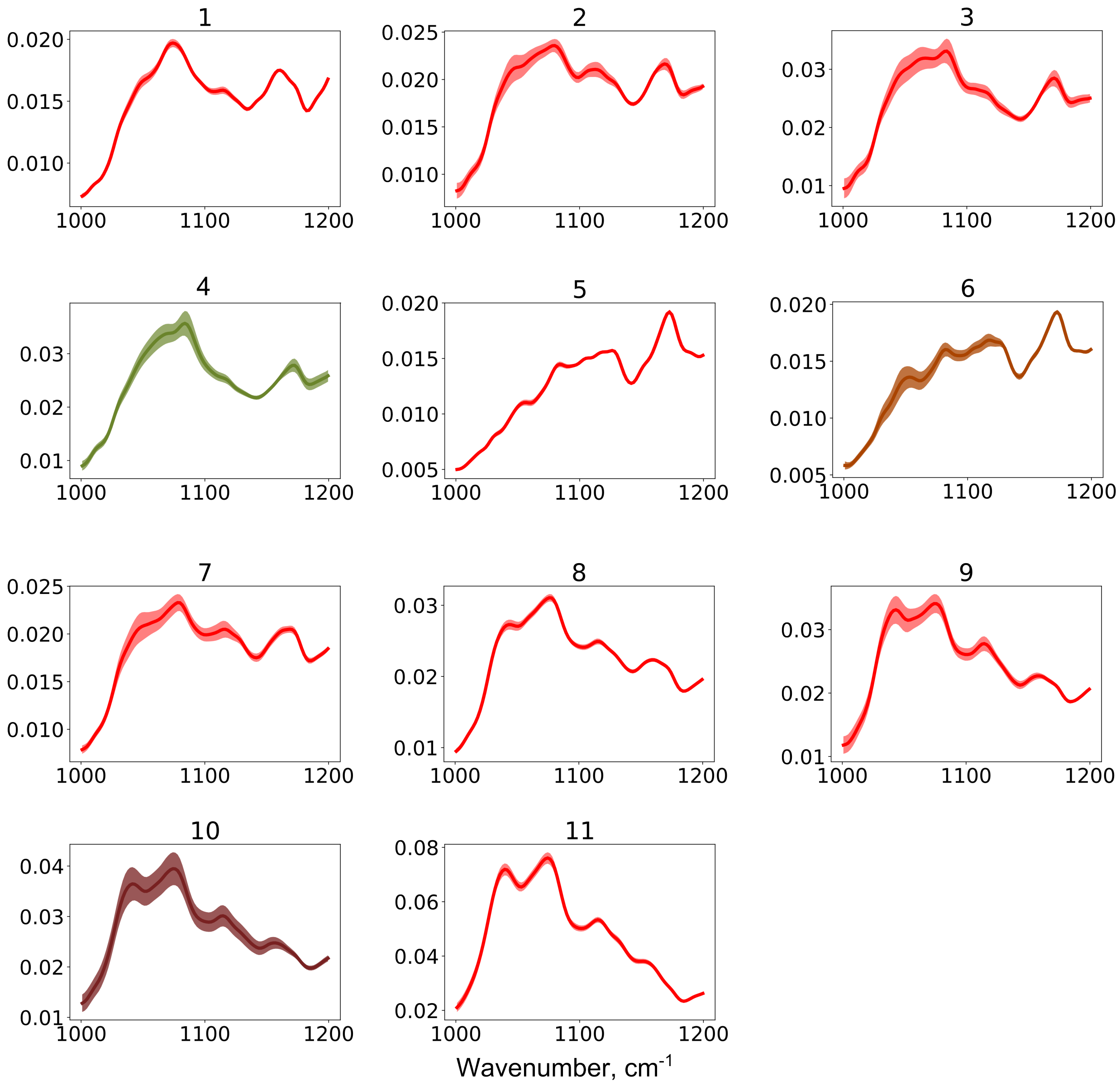

Figure S9: Between-person variability of the infrared spectra of protein fractions in the carbohydrate region, measured in a group of 25 healthy volunteers.

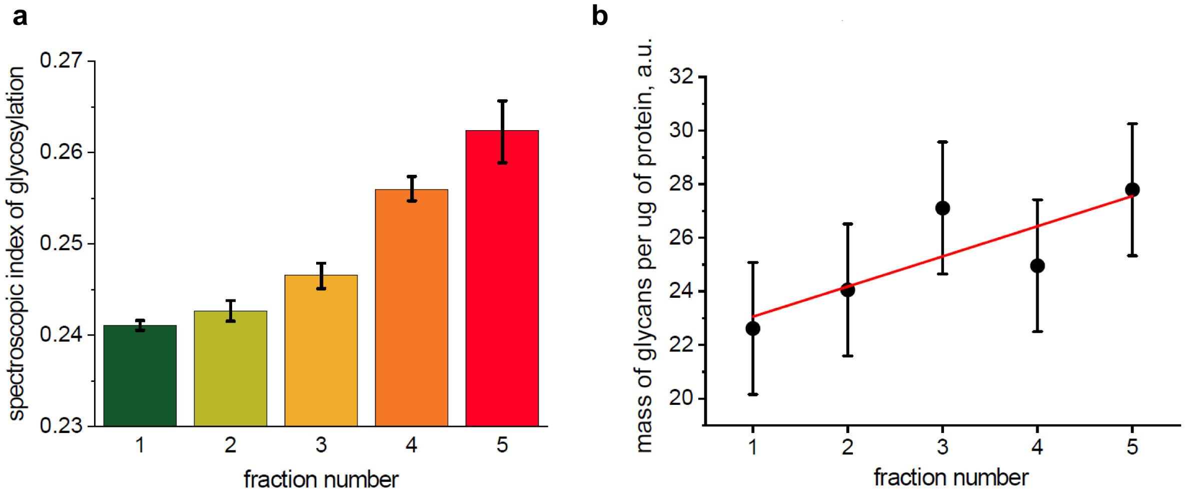

Figure S10: The change in the overall level of glycosylation of alpha-1-acid glycoprotein proteoforms with elution time determined by (a) infrared spectroscopy; (b) MS-based glycomics.

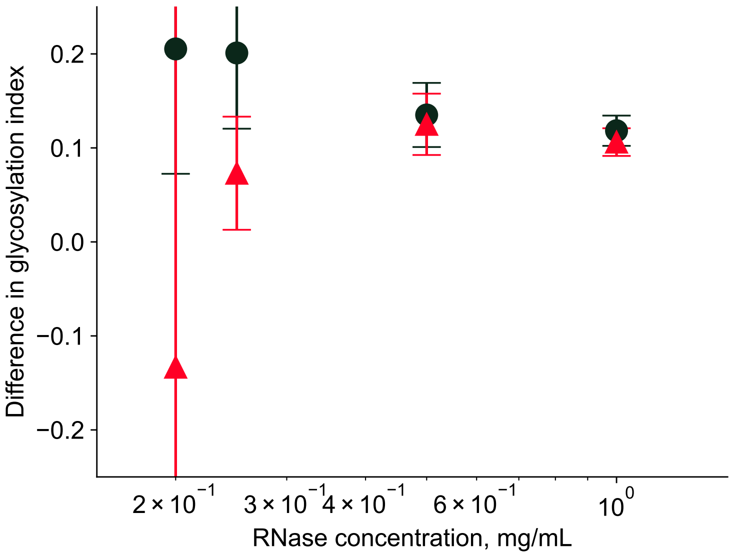

Figure S11: Difference between spectroscopic global glycosylation indexes of RNase B and RNase A determined in pure water (black dots) and after spiking into human blood plasma and IEX separation (red triangles). Dividing the difference in spectroscopic global glycosylation indexes plotted here by the error bar produces the effect size that is plotted in Figure 4 of the main text.

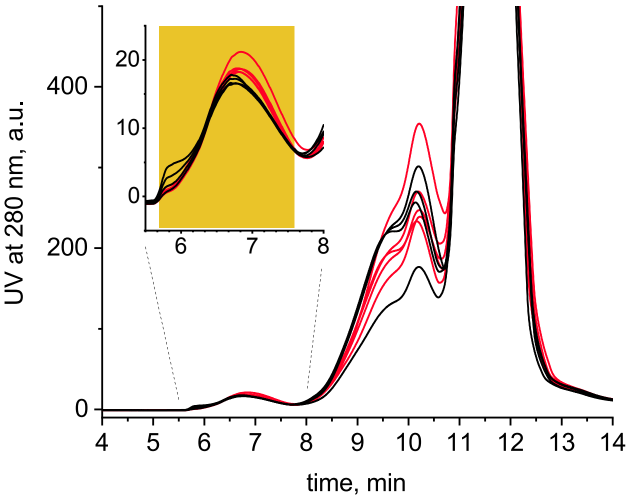

Figure S12: Chromatographic separation of blood plasma samples with 1 mg/mL RNase spiked to them. The peak corresponding to the RNase is enlarged. Black lines - RNase A is spiked, red lines - RNase B. For further analysis the fraction from 5.7 to 7.6 mins was collected - shown in yellow. Note that the majority of plasma proteins elute as two broad features because the gradient was modified to decrease the analysis time (see Figure SS5 compared to Figure SS2).

Table S2: The composition of protein fractions after IEX separation measured via MS-based proteomics.

Fraction	Protein	% of total mass
1	IgG	92
	Apolipoprotein H	5
	Other	3
2	Serotransferrin	72
	Hemopexin	11
	Other	17
3	Serotransferrin	34
	Hemopexin	20
	Apolipoproteins	19
	Fibrinogen	12
	Other	16
4	Apolipoproteins	40
	Fibrinogen	22
	Hemopexin	12
	Serotransferrin	7
	Other	19
5	Serum albumin	75
	Apolipoproteins	13
	Other	12
6	Serum albumin	53
	IgA	10
	Apolipoproteins	10
	Complement C3	8
	Vitamin D-binding protein	8
	Other	11
7	Serum albumin	31
	IgA	20
	Apolipoproteins	9
	Alpha-2-HS-glycoprotein	6
	Other	34
8	Alpha-1-antitrypsin	18
	IgA	14
	Alpha-2-HS-glycoprotein	10
	Transthyretin	9
	Serum albumin	7

	Apolipoproteins	7
	Haptoglobin	6
	Other	29
9	Haptoglobin	24
	IgA	11
	Alpha-2-HS-glycoprotein	8
	Serum albumin	8
	Ceruloplasmin	7
	Retinol-binding protein	7
	Transthyretin	6
	Apolipoproteins	5
	Other	23
10	Haptoglobin	30
	Ceruloplasmin	8
	Apolipoproteins	7
	IgA	5
	Other	50
11	Orosomucoid	65
	Other	35

Table S3: The most abundant glycan compositions and the masses of corresponding ions observed in each chromatographic peak for human alpha-1-acid glycoprotein. Glycan compositions are expressed as: Nx, number (x) of N-Acetylhexosamines; Hx, number (x) of Hexoses; Sx, number (x) of N-Acetylneuraminic acids; Fx, number (x) of Fucoses.

Glycan peak	Theoretical mass $[M+H]^+$	Theoretical mass $[M+2H]^{2+}$	Peak composition
1	1860.781	930.894	N4H5
2	2006.755	1003.923	N4H5F1
	1989.742	995.415	N4H4S1
3	2151.877	1076.442	N4H5S1
4	2151.877	1076.442	N4H5S1
5	2297.934	1149.471	N4H5S1F1
6	2517.009	1259.008	N5H6S1
	2442.972	1221.99	N4H5S2
7	2442.972	1221.99	N4H5S2
8	2663.067	1332.037	N5H6S1F1
9	2589.03	1295.019	N4H5S2F1
10	2808.104	1404.556	N5H6S2

11	2882.141	1441.574	N6H7S1
	2808.104	1404.556	N5H6S2
12	2808.104	1404.556	N5H6S2
13	2954.162	1477.585	N5H6S2F1
14	3099.2	1550.103	N5H6S3
	3173.236	1587.122	N6H7S2
15	3099.2	1550.103	N5H6S3
16	3173.236	1587.122	N6H7S2
17	3245.257	1623.132	N5H6S3F1
	3464.332	1732.669	N6H7S3
18	3099.2	1550.103	N5H6S3
19	3245.257	1623.132	N5H6S3F1
20	3464.332	1732.669	N6H7S3
	3245.257	1623.132	N5H6S3F1
21	3464.332	1732.669	N6H7S3
22	3464.332	1732.669	N6H7S3
	3610.39	1805.698	N6H7S3F1
23	3755.427	1878.217	N6H7S4
	3610.39	1805.698	N6H7S3F1
24	3755.427	1878.217	N6H7S4
	3610.39	1805.698	N6H7S3F1
25	3755.427	1878.217	N6H7S4
	3901.485	1951.246	N6H7S4F1
26	3755.427	1878.217	N6H7S4
	3901.485	1951.246	N6H7S4F1
27	3901.485	1951.246	N6H7S4F1
	3755.427	1878.217	N6H7S4
28	3901.485	1951.246	N6H7S4F1
	3829.464	1915.236	N7H8S3
29	3901.485	1951.246	N6H7S4F1
	4047.543	2024.275	N6H7S4F2
30	4120.559	2060.783	N7H8S4
31	4120.559	2060.783	N7H8S4
	4194.596	2097.802	N8H9S3
32	4266.617	2133.812	N7H8S4F1
	4120.559	2060.783	N7H8S4

33	4485.692	2243.349	N8H9S4
34	4485.692	2243.349	N8H9S4
	4793.813	2397.41	N8H9S5
35	4793.813	2397.41	N8H9S5
	4939.871	2470.439	N8H9S5F1
